# Efficacy of the low dose apatinib plus deep hyperthermia as third-line or later treatment in HER-2 negative advanced gastric cancer

**DOI:** 10.3389/pore.2023.1611114

**Published:** 2023-07-03

**Authors:** Guohu Han, Changchun Sun, Lihua Cui, Yufeng Huang, Lijiang Yu, Shenzha Liu, Min Tao

**Affiliations:** ^1^ Department of Oncology, Dushu Lake Hospital Affiliated to Soochow University, Suzhou, Jiangsu, China; ^2^ Department of Oncology, Jingjiang People’s Hospital, The Seventh Affiliated Hospital of Yangzhou University, Jingjiang, Jiangsu, China; ^3^ Department of Oncology, The First Affiliated Hospital of Soochow University, Suzhou, Jiangsu, China

**Keywords:** apatinib, deep hyperthermia, gastric cancer, efficacy evaluation, prognosis

## Abstract

**Aim:** To observe the efficacy of the low dose apatinib plus deep hyperthermia as third-line or later treatment for patients with human epidermal growth factor receptor 2 (HER-2) negative advanced gastric cancer.

**Methods:** 80 eligible patients with HER-2 negative advanced gastric cancer admitted to Jingjiang People’s Hospital Affiliated with Yangzhou University-from March 2021 to March 2022 were selected, and they were divided into the control group (*n* = 40, apatinib) and experimental group (*n* = 40, apatinib plus deep hyperthermia) on the basis of random number table method. The levels of serum carcinoembryonic antigen (CEA), carbohydrate antigen 199 (CA199), and vascular endothelial growth factor (VEGF) were monitored, and the efficacy of the two groups was analyzed by referring to Karnofsky performance status (KPS), overall survival (OS) and disease control rate (DCR) before and after treatment.

**Results:** The levels of CEA, CA199, and VEGF in both groups were lower after treatment than before (*p* < 0.05), and lower (CEA: 8.85 ± 1.36 vs. 12.87 ± 1.23, CA199: 34.19 ± 4.68 vs. 50.11 ± 5.73, VEGF: 124.8 ± 18.03 vs. 205.9 ± 19.91) in the experimental group than in the control group (*p* < 0.05). The DCR and KPS of the patients in the experimental group were significantly higher (DCR: 62.50% vs. 40.00%; KPS: 83.25 ± 1.15 vs. 76.25 ± 1.17) than in the control group (*p* < 0.05). In survival analysis, patients with control group had shorter OS than the experimental group. (median 5.65 vs. 6.50 months; hazard ratio [HR], 1.63 [95% confidence interval (CI) 1.02–2.60], *p* = 0.0396).

**Conclusion:** The application of low-dose apatinib plus deep hyperthermia for patients with HER-2 negative gastric cancer who failed second-line treatment should be a promising option.

## Introduction

Advanced gastric cancer is one of the common malignant tumors in the gastrointestinal tract or gastrointestinum, which presents a high mortality rate and poses a threat to human public health [1–3]. The absolute majority of patients have entered the advanced stage and lost the opportunity for surgery once diagnosed, and it is difficult to treat [4, 5]. For these patients, the first- and second-line treatment is mainly based on platinum, fluorouracil, and taxane-based chemotherapy [6]. However, due to the low efficacy and side effects of chemotherapy, researchers have been considering trying new drugs. Under such a circumstance, apatinib is a novel antiangiogenic agent that can highly selectively inhibit vascular endothelial growth factor (VEGF) receptor and block tumor angioneogenesis to achieve antitumor effect [7].

In recent years, deep hyperthermia as a new type of antitumor treatment has gradually become another tumor treatment method after surgery, chemotherapy, and targeted therapy [8]. Because of its safety, effectiveness and low adverse reactions, it may produce synergistic effects when added to conventional therapy [8, 9]. However, no studies have definitively determined the efficacy of deep hyperthermia and apatinib in advanced gastric cancer. In the present study, we intend to explore the therapeutic effect to provide references for patients with human epidermal growth factor receptor 2 (HER-2) negative gastric cancer who have failed second-line treatment.

## Materials and methods

### Baseline data

80 patients with HER-2 negative gastric cancer who had failed second-line treatment in our hospital from March 2021 to March 2022 were selected and were divided into two groups. Before enrollment, all the clinical baseline information of patients was relatively balanced in terms of sex, age, site of primary tumor, histology and number of prior chemotherapy regimens between the two groups (all *p* > 0.05), as shown in Table 1. This study had been approved by the Ethics Committee of Jingjiang People’s Hospital Affiliated with Yangzhou University (No. 2021-03-029). All subjects in this study provided their written informed consent consistent with the Declaration of Helsinki.

**TABLE 1:** Baseline patient characteristics (n (%)).

Characteristic	Control group (*n* = 40)	Experimental group (*n* = 40)	χ^2^	*P*
Sex				
Male	25 (62.50%)	28 (70.00%)	0.503	0.478
Female	15 (37.50%)	12 (30.00%)		
Age (years)				
<65	23 (57.50%)	21 (52.50%)	0.202	0.653
≥65	17 (42.50%)	19 (47.50%)		
Site of primary tumor				
Fundus	9 (22.50%)	6 (15.00%)	1.533	0.675
Body	9 (22.50%)	11 (27.50%)		
Antrum	16 (40.00%)	14 (35.00%)		
Cardia	6 (15.00%)	9 (22.50%)		
Histology				
Well differentiated	0 (0.00%)	1 (2.50%)	1.671	0.434
Moderately differentiated	21 (52.50%)	24 (60.00%)		
Poorly differentiated	19 (47.50%)	15 (37.50%)		
Number of prior chemotherapy regimens				
2	29 (72.50%)	31 (77.50%)	0.267	0.606
≥3	11 (27.50%)	9 (22.50%)		

### Inclusion and exclusion criteria

Inclusion criteria were as follows: [1] patients confirmed to have HER-2 negative advanced gastric cancer, and the metastatic disease was confirmed by clinical, imaging, histological, or cytological measures. [2] patients who failed after second-line treatment [3], patients with expected survival time greater than 3 months [4], patients with sound heart, lung, liver and kidney functions [5], patients with no second primary tumor, and [6] patients who voluntarily provided informed consent. Exclusion criteria were as follows: [1] patients with hypertension (systolic blood pressure>140 mm Hg, diastolic blood pressure>90 mm Hg) that could not be reduced to the normal range by antihypertensive drug treatment [2], patients with coagulation dysfunction [3], patients with disease [4], patients who did not coordinate with clinical follow-up [5], patients with a clear tendency to gastrointestinal bleeding, and [6] patients with incomplete clinical and pathological data.

### Treatment methods

Control group: patients received 250 mg/d of apatinib (Jiangsu Hengrui Medicine Co., Ltd., SFDA approval No. H20140103, 0.25 g) orally. Experimental group: on the basis of apatinib regimen in the control group, patients received deep hyperthermia (Nanjing Hengpu Weiye Technology Co., LTD, NO. HY7000-1). The method of deep hyperthermia: before hyperthermia, the location and size of the tumor should be determined according to the results of CT, B-ultrasound or MRI examination. The tumor should be taken as the central point for positioning, appropriate plate and body position should be selected, and water bags with appropriate size and temperature should be selected according to the individual situation of the patient. The patient was supine. After adjusting the position, a water bag was placed on the skin of the corresponding treatment site (covering the treatment site), and the plate was lowered to make it close to the water bag. The treatment was carried out under the computer monitoring. Deep hyperthermia device with a power of 400–800 W for twice a week, and a preset temperature of 42°C–43°C, and each treatment lasted for 30 min, and each deep hyperthermia was performed within 1 h after oral apatinib. Antitumor treatment was 8 consecutive weeks long.

### Outcome measures

Tumor biomarkers determination: Sera were obtained through centrifuging fasting venous blood, employing an automatic electrochemiluminometer E170® and assorted kits (Roche™, Switzerland). Serum free vascular epidermal growth factor (VEGF) was detected by ELISA kit (R&D Systems Inc., Minneapolis, MN, USA) according to the instructions. References ranged as follows: CEA <3.5 ng/mL, CA199 <39 U/Ml, VEGF: 6.25–142.2 pg/mL.

The efficacy was assessed by referring to Response Evaluation Criteria In Solid Tumors (RECIST) [6]. Complete remission (CR): all targeted lesions disappeared without new ones were found for more than 1 month; partial remission (PR): the total diameter of the target lesions was decreased in volume at least 30%, and no new lesions were found for more than 1 month; progressive disease (PD): the total diameter of the target lesions increased at least 20%, or new lesions were found; stable disease (SD): Neither sufficient shrinkage to qualify PR nor sufficient increase to qualify for PD; disease control rate (DCR)= (number of CR cases+ number of PR cases+ number of SD cases)/total number of cases × 100%. The clinical outcome of OS was calculated as the time from the first treatment to the date of death.

The percentage of the Karnofsky performance status (KPS) was used to describe the physical health by three conditions: A (0%–40%), B (50%–70%), and C (80%–100%). KPS scores range from 0 to 100, and 100 is fully capable of in daily activities without clinical evidence of disease (signs or symptoms), 0 means death (Table 2).

**TABLE 2:** Karnofsky performance status.

Conditions	Percentage	Comments
A: Able to perform normal daily activities and work. No special care is necessary	100	Normal, without symptoms or signs of disease
	90	Ability to perform normal daily activities, minor symptoms or signs of disease
	80	perform normal daily activities with some effort, some symptoms or signs of disease
B: Ability to live at home and take care of most personal needs. Assistance is needed to varying degrees	70	Can take care of self, unable to do work or maintain normal daily activities. Such as cooking, playing football, and driving a car
	60	Can take care of self the most of the time, but occasionally required considerable assistance
	50	Required considerable assistance frequently
C: Unable to take care of self. Hospital care is required. Disease can advance rapidly	40	Disabled, requires medical care and assistance, in bed ≥50% of the time
	30	Seriously disabled, unable to take care of themselves, almost always in bed
	20	Seriously bedfast, hospitalization necessary, requiring active supportive treatment
	10	Moribund, comatose or difficult to wake up
	0	Dead

The adverse reactions were graded by the National Cancer Institute’s Common Terminology Criteria for Adverse Events version 4.0 (NCI-CTCAE v4.0).

### Statistical analysis

SAS version 9.2 (SAS Institute, Inc., Cary, NC) and GraphPad Prism 8.02 (GraphPad Software, Inc.) software were performed for statistical analyses. Fisher’s exact test or chi-square test, correction of continuity chi-square test were used to compare categorical data for baseline characteristics and adverse reactions between the control group and the experimental group. The continuous data were presented by mean ± standard deviations (mean ± SD), and the comparison of the mean ± SD before and after treatment in the same group was performed by paired samples *t*-test, and the comparison of the mean ± SD of the two groups was performed by Unpaired independent samples *t*-test. Overall survival description was illustrated by the Kaplan-Meier curves, with *p*-value determined by a log-rank test, and the 95% CI for the median time to each event was computed. *p* < 0.05 was considered indicating a statistically significant difference (2-sided).

## Results

### Comparison of the levels of CEA, CA199, and VEGF before and after treatment between the two groups

After treatment, the levels of CEA, CA199, and VEGF were significantly reduced than before treatment in the control group (CEA: 12.87 ± 1.23 vs. 15.79 ± 0.81, CA199: 50.11 ± 5.73 vs. 93.59 ± 8.62, VEGF: 205.9 ± 19.91 vs. 292.8 ± 11.90) and experimental group (CEA: 8.85 ± 1.36 vs. 16.58 ± 1.14, CA199: 34.19 ± 4.68 vs. 89.45 ± 3.03, VEGF: 124.8 ± 18.03 vs. 299.6 ± 11.39). After treatment, the levels of these indicators in the experimental group were significantly lower (CEA: 8.85 ± 1.36 vs. 12.87 ± 1.23, CA199: 34.19 ± 4.68 vs. 50.11 ± 5.73, VEGF: 124.8 ± 18.03 vs. 205.9 ± 19.91) than in the control group (all *p* < 0.05, Tables 3–5).

**TABLE 3:** Comparison of serum CEA expression level (mean ± standard deviation).

Groups	*n*	Before treatment	After treatment	*T*	*P*
Control group	40	15.79 ± 0.81	12.87 ± 1.23	2.818	0.008
Experimental group	40	16.58 ± 1.14	8.85 ± 1.36	3.538	0.001
*t*		0.569	2.199		
*P*		0.571	0.031		

Note: CEA, carcinoembryonic antigen (μg/L).

**TABLE 4:** Comparison of serum CA199 expression level (mean ± standard deviation).

Groups	*n*	Before treatment	After treatment	*t*	*P*
Control group	40	93.59 ± 8.62	50.11 ± 5.73	3.524	0.001
Experimental group	40	89.45 ± 3.03	34.19 ± 4.68	8.705	<0.001
*t*		0.453	2.153		
*P*		0.652	0.034		

Note: CA199, carbohydrate antigen 199, (U/Ml).

**TABLE 5:** Comparison of serum VEGF expression level (mean ± standard deviation).

Groups	*n*	Before treatment	After treatment	*t*	*P*
Control group	40	292.8 ± 11.90	205.9 ± 19.91	3.382	0.002
Experimental group	40	299.6 ± 11.39	124.8 ± 18.03	6.719	<0.001
*t*		0.416	3.018		
*P*		0.679	0.003		

Note: VEGF, Vascular endothelial growth factor, (pg/mL).

### Comparison of the percentages of the Karnofsky performance status (KPS) and incidence of adverse reactions before and after treatment between the two groups

After treatment, the percentages of the KPS were significantly increased than before treatment in the control group (76.25 ± 1.17 vs. 71.00 ± 1.67) and experimental group (83.25 ± 1.15 vs. 72.75 ± 1.64). After treatment, the percentage of the KPS in the experimental group was significantly higher (83.25 ± 1.15 vs. 76.25 ± 1.17) than in the control group (all *p* < 0.05, Table 6). After treatment, there were no statistical significance in the incidence of adverse reactions between the control group and the experimental group (all *p* > 0.05, Table 7).

**TABLE 6:** Comparison of KPS scores (mean ± standard deviation).

Groups	*n*	Before treatment	After treatment	*t*	*P*
Control group	40	71.00 ± 1.67	76.25 ± 1.17	3.667	0.001
Experimental group	40	72.75 ± 1.64	83.25 ± 1.15	9.802	<0.001
*T*		0.747	4.259		
*P*		0.457	<0.001		

Note: KPS, Karnofsky performance status.

**TABLE 7:** Comparison of adverse reactions of patients (n (%)).

Groups	*n*	Hypertension	Albuminuria	Bone marrow suppression	Diarrhea
Control group	40	18 (45.00%)	11 (27.50%)	15 (37.50%)	18 (45.00%)
Experimental group	40	15 (37.50%)	9 (22.50%)	13 (32.50%)	21 (52.50%)
χ^2^		0.464	0.267	0.220	0.450
*P*		0.496	0.606	0.639	0.502

Note: Bone marrow suppression: Neutropenia or Anemia or Thrombocytopenia.

### Comparison of clinical efficacy before and after treatment between the two groups

**FIGURE 1:**
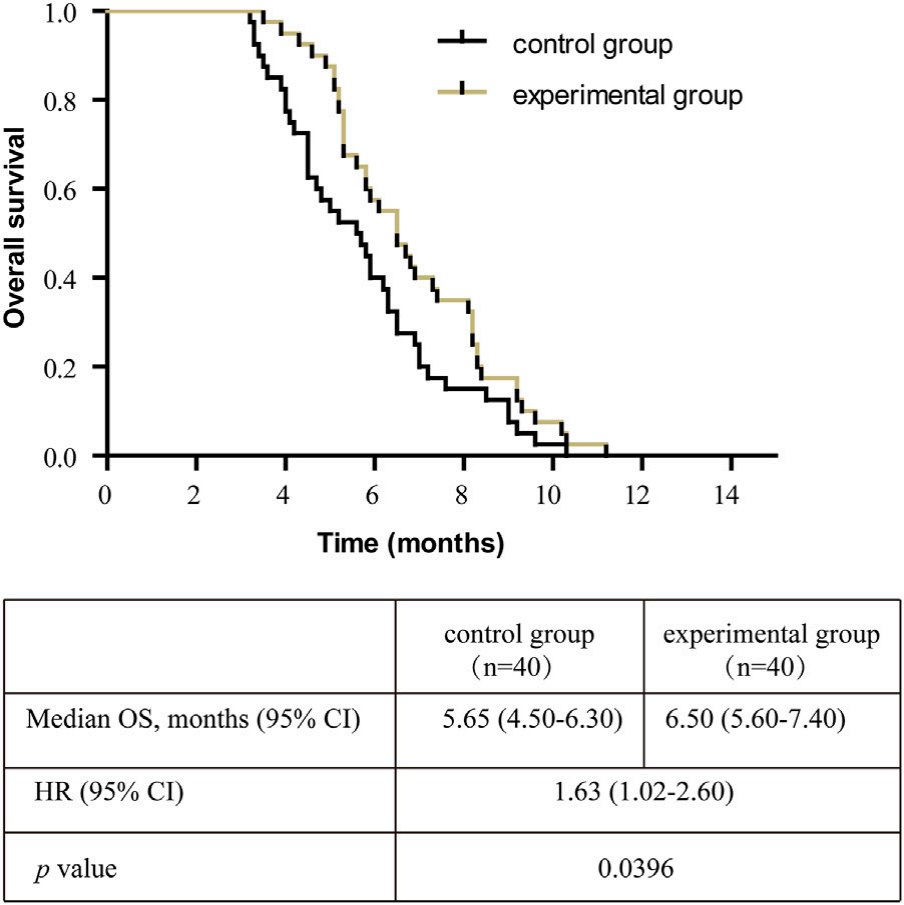
Kaplan-Meier survival curve of the overall survival (OS) in all patients. Note: OS, overall survival; CI, confidence interval; HR, hazard ratio.

After treatment, the experimental group was superior to the control group in the matter of DCR (62.50% 25/40% vs. 40.00% 16/40) (*p* < 0.05, Table 8). The median OS of the control group and experimental group were 5.65 months (95% CI, 4.50–6.30) vs. 6.50 months (95% CI, 5.60–7.40) respectively, and the OS in the control group was shorter than the experimental group (*p* = 0.0396, Figure 1).

**TABLE 8:** Comparison of clinical efficacy (n (%)).

Groups	Control group (*n* = 40)	Experimental group (*n* = 40)	χ^2^	*P*
CR	0 (0.00%)	0 (0.00%)		
PR	3 (7.50%)	8 (20.00%)		
SD	13 (32.50%)	17 (42.50%)		
PD	24 (60.00%)	15 (37.50%)		
DCR	16 (40.00%)	25 (62.50%)	4.053	0.044

Note: CR, complete response; PD, progressive disease; PR, partial response; SD, stable disease; DCR, disease control rate.

## Discussion

Gastric cancer has become one of the malignant tumors that has impacted on human life span [1, 2]. Despite its worldwide decline in incidence over the past century, gastric cancer remains a major killer across the globe [10]. For the treatment of patients with advanced gastric cancer, the development from traditional chemotherapy to molecular precision targeted therapy has extended the survival period of patients and improved the quality of life [11]. The principle of hyperthermia is to utilize the thermal sensitivity of tumor cells to increase the permeability of the membrane structure of tumor cells so that the concentration of anti-tumor drugs in the tumor increases, and the death of tumor cells is accelerated, but the normal tissue around the lesion will not be damaged because of its normal blood flow and good heat dissipation [12].

Apatinib, a novel receptor tyrosine kinase inhibitor that highly selectively inhibits the binding of VEGF and vascular epidermal growth factor-2 (VEGFR-2), blocking the activation of mitogen-activated protein kinase (MAPK) signaling pathway, achieving the purpose of anti-tumor neovascularization and preventing further proliferation and metastasis of tumor cells [13]. Since 2014, apatinib has been approved by Chinese Society of Clinical Oncology (CSCO) guideline as a third-line or follow-up treatment for advanced gastric cancer and gastroesophageal junction adenocarcinoma in China [14], providing an important basis for clinical medication guidance. For the third-line treatment of patients with advanced gastric cancer, although apatinib is recommended by the guidelines, there are still some patients who refuse to receive apatinib, because of potential side effects requiring dietary restriction, severe hypertension with poor drug control, as well as intolerance to apatinib [15–17]. Therefore, low-dose apatinib was selected in this study for patients who had failed second-line and late treatment.

A previous study showed a highly positive correlation between the level of VEGF and tumor progression of patients with gastric cancer [18]. VEGF was highly expressed in gastric cancer, closely related to TNM staging and lymph node metastasis, which predicted worse prognosis of gastric cancer patients [19]. In addition to VEGF, serum CEA is a well-characterized glycoprotein, which is associated with depth of tumor invasion, lymph node metastasis and tumor metastasis, and is usually used to diagnose gastrointestinal malignancies and monitor of therapeutic effects in gastric cancer, esophageal cancer and breast cancer [20, 21]. Raised serum CA199 level was positively related to poor prognosis, tumor size, metastasis and invasion in gastric cancer patients [20, 21]. Nevertheless, the merge effect of these tumor markers on apatinib plus deep hyperthermia in advanced gastric cancer is unclear. In our study, the results showed that after treatment, the levels of serum CEA, CA199, and VEGF were significantly reduced than before treatment in the control group and experimental group. At the same time, compared with single apatinib, the levels of these indicators in the experimental group were much lower. Thus, deep hyperthermia plus apatinib may become a novel strategy for the treatment of advanced gastric cancer.

In a real-world study, 747 patients who had failed at least second-line therapy treated with low-dose apatinib (250 or 500 mg/d). Four patients achieved CR, 47 achieved PR, and 374 achieved SD. The DCR was 56.89% [22]. Moreover, following the result of a previous meta-analysis: concerning DCR, apatinib (odds ratio 7.84, 95% CI 4.12–16.50) was the best treatment for the third-line treatment of advanced gastric cancer in contrast to the third-line chemotherapy [23]. The study suggests that low dose apatinib is an effective treatment for advanced gastric cancer. Hyperthermia in advanced gastric cancer improved survival and clinical outcomes (DCR), and reduced recurrence according to a meta-analysis of studies [24]. Hyperthermia can enhance the chemotherapeutic efficacy of cisplatin-based therapy in gastric cancer [25]. A recent study showed that 500 mg of apatinib combined with chemotherapy obtained significantly higher DCR and KPS scores of compared with the chemotherapy group (*p* < 0.05) [26]. In this study, after treatment, the experimental group was superior to the control group in the matter of DCR, OS and KPS. The incidence of adverse reactions was similar between the two groups, and the overall tolerance was good. Although this study did not find statistically significant differences in the incidence of the above adverse reactions between the two groups, it still needs clinical attention. The results showed that apatinib plus deep hyperthermia displayed a synergistic effect. Its mechanism of action is that apatinib accelerates the thermal sensitivity of tumor cells. After the tumor tissue is heated, the gene expression and protein synthesis of VEGF are significantly inhibited, inhibiting the proliferation of endothelial cells and the formation of tumor neovascularization, while increasing the permeability of the cell membrane, making it easy for drugs to enter the tumor cells, and maintaining a high concentration of drugs in the cells, thereby improving the killing effect of tumors [27, 28]. On the other hand, Apatinib inhibits neovascularization and reduces repair ability of tumor tissue; Moreover, high-temperature causes protein denaturation and coagulative necrosis of local tumor cells, causing tumor tissue to fall off, affecting the synthesis and repair function of tumor cells, thus damaging cells and achieving the purpose of controlling tumors [29, 30]. Although numerous studies have continuously confirmed the clinical value of deep hyperthermia, the current clinical technology of hyperthermia is poor, and accurate deep hyperthermia cannot be realized, sometimes it has little effect, which leads to some scholars skeptical of tumor hyperthermia.

The features and innovations of this study are as follows [1]: Through comparative analysis of apatinib combined with deep hyperthermia, to explore the feasibility of combined therapy [2]; By comparing the total effective rate and quality of life of different subjects, the excellent performance of apatinib combined with deep hyperthermia in the treatment of advanced gastric cancer was discussed, and the clinical significance and application value of the above therapy for advanced gastric cancer was generally evaluated, revealing the prospect of clinical application. Although there are different opinions on the third-line treatment for patients with advanced gastric cancer, our study is designed only to explore an effective treatment method. Our study is the first to use apatinib plus deep hyperthermia in the treatment of gastric cancer, so some limitations that still merit attention.

This study had several limitations. First, the effective sample capacity was small owing to the study included only 80 patients. Second, long-term complications, such as hoarseness and hand-foot syndrome, were not assessed because of the short follow-up period and short treatment cycle. Third, no observation of progression free survival time, further verification of multi-center, large-sample clinical trial is still needed in the future.

## Conclusion

In conclusion, apatinib with deep hyperthermia is superior to apatinib alone in advanced gastric cancers cases, which can effectively reduce the levels of tumor markers, significantly improve clinical response rate, enhance the quality of life, demonstrate longer OS in patients. Besides, deep hyperthermia is a non-invasive therapy, and patients are psychologically willing to accept it with good compliance, which is worth promoting diligently.

## Data Availability

The original contributions presented in the study are included in the article/supplementary material, further inquiries can be directed to the corresponding authors.
